# GSK-3α/β and MEK inhibitors assist the microenvironment of tumor initiation

**DOI:** 10.1007/s10616-023-00575-1

**Published:** 2023-04-01

**Authors:** Ghmkin Hassan, Said M. Afify, Maram H. Zahra, Hend M. Nawara, Kazuki Kumon, Yoshiaki Iwasaki, David S. Salomon, Akimasa Seno, Masaharu Seno

**Affiliations:** 1 Department of Cancer Stem Cell Engineering, Faculty of Interdisciplinary Science and Engineering in Health Systems, Institute of Academic and Research, Okayama University, Okayama, 700-8530 Japan; 2 Division of Biochemistry, Chemistry Department, Faculty of Science, Menoufia University, Shebin El Koum, Menoufia 32511 Egypt; 3 Research Core for Interdisciplinary Sciences, Graduate School of Natural Science and Technology, Okayama University, Tsushima Naka, Kita, Okayama, 700-8530 Japan; 4 Health Service Center, Okayama University, Okayama, 700-8530 Japan; 5 Center for Cancer Research, National Cancer Institute, Frederick, MD 21702 USA; 6 The Laboratory of Natural Food and Medicine, Co, Ltd., Okayama, 700-8530 Japan; 7 R&D Center, Katayama Chemicals Ind., Co. Ltd, 4.1.7 Ina, Minoh, Osaka, 562-0015 Japan; 8 Department of Oncology, Lombardi Comprehensive Cancer Centre, Georgetown University, Washington, DC 20007 USA; 9 Department of Microbiology and Biochemistry, Faculty of Pharmacy, Damascus University, Damascus, Syria

**Keywords:** Cancer stem cells, Human iPSCs, Signal pathway inhibitors, Tumor initiation

## Abstract

Induced pluripotent stem cells (iPSCs) are useful tools for modeling diseases and developing personalized medicine. We have been developing cancer stem cells (CSCs) from iPSCs with conditioned medium (CM) of cancer-derived cells as the mimicry of the microenvironment of tumor initiation. However, the conversion of human iPSCs has not always been efficient with only CM. In this study, human iPSCs reprogrammed from monocytes of healthy volunteers were cultured in a media containing 50% of the CM from human pancreatic cancer derived BxPC3 cells supplemented with a MEK inhibitor (AZD6244) and a GSK-3α/β inhibitor (CHIR99021). The survived cells were assessed for the characteristics of CSCs in vitro and in vivo. As a result, they exhibited CSC phenotypes of self-renewal, differentiation, and malignant tumorigenicity. Primary culture of the malignant tumors of the converted cells exhibited the elevated expression of CSC related genes CD44, CD24 and EPCAM maintaining the expression of stemness genes. In conclusion, the inhibition of GSK-3α/β and MEK and the microenvironment of tumor initiation mimicked by the CM can convert human normal stem cells into CSCs. This study could provide insights into establishing potentially novel personalized cancer models which could help investigate the tumor initiation and screening of personalized therapies on CSCs.

**Supplementary Information:**

The online version contains supplementary material available at 10.1007/s10616-023-00575-1.

## Introduction

Cancer stem cells (CSCs) are highly tumorigenic subpopulations of cancer cells with self-renewal ability. CSCs are responsible for tumor initiation and CSC microenvironment is thought to play a crucial role in tumor initiation and progress (Bayik and Lathia 2021). The impact of the inflammation and tissue repair process on cancer development has gained enormous interest in recent decades. Chronic inflammation generates imbalanced microenvironment and could convert normal stem cells residing in tissues into CSCs as a trigger of cancer initiation (Coussens and Werb 2002; Wang et al. 2017). The conversion of normal stem cells into CSCs under the imbalanced microenvironment should be involved with altered activation of intracellular signaling pathway. The converted cells will grow and develop tumors if they can escape immune defenses and had proper microenvironment supporting their stemness (Beatty and Gladney 2015).

Recently, many efforts have been paid to generate CSC models from induced pluripotent stem cells (iPSCs) for different types of cancers by numerous approaches. Although, these models could be used as tools to investigate tumorigenesis and design new therapies applicable to CSCs, the exact mechanism of generation of CSCs still unknown (Chao and Chern 2018).

In this context, our lab has developed a novel method to generate CSCs from iPSCs under a cancer initiating microenvironment using conditioned media (CM) from different cancer cells. Mouse iPSCs were used to generate pancreatic, liver, lung and breast CSC models using this method (Afify et al. 2020; Calle et al. 2016; Chen et al. 2012). In a previous study, we converted mouse iPSCs (miPSCs) into CSCs using CM of Lewis lung carcinoma (LLC) cells and non-mutagenic chemical compounds. The chemical compounds inhibiting different signaling inhibition activity accelerated the conversion of mouse iPSCs into CSCs. Among these inhibitors, a glycogen synthase kinase (GSK) 3β inhibitor CHIR99021, and a MEK inhibitor PD0325901 were found to significantly enhance the conversion process. The inhibition of MAPK/ERK pathway and overactivation of PI3K-AKT pathway were suggested as a mechanism of CSC induction (Du et al. 2020). Recently, we reported that among different MAPK cascade inhibitors, the AZD6244 (Selumetinib), which was an inhibitor of MEK1, was the most effective inhibitor in the conversion of miPSCs into CSCs (Hassan et al. 2021).

A combination of CHIR99021 and PD0325901 was shown to be effective in the maintenance of the naïve state of embryonic stem cells in serum-free media where inhibition of the MAPK pathway had a significant role in the effect (Sim et al. 2017; Song et al. 2017). Thus, here we tested whether a combination of CM from cancer cells, CHIR99021 and AZD6244 would induce the conversion of human iPSCs (hiPSCs) into CSCs. In this study, the microenvironment of tumor initiation was mimicked by culturing hiPSCs in CM from a human pancreatic cancer cell line BxPC3 cells, AZD6244 and CHIR99021 for one month. The converted cells were evaluated for CSC characteristics in vitro and in vivo. The converted cells developed malignant tumors when injected into immunodeficient mice and the primary cells isolated from tumors exhibited CSC phenotype.

## Material s and methods

### Cell culture

The blood samples were obtained from two healthy volunteers under the approval No. KEN1812-032 from the ethics committee of Okayama University Hospital. And the monocytes were purified with BD Vacutainer CPT (REF 362,753, Becton Dickinson and Company, NJ) and reprogrammed to iPSCs with Sendai virus vector, SeVdp (KOSM)302L which was a kind gift from Dr. Mahito Nakanishi, the National Institute of Advanced Industrial Science and Technology (AIST), Tsukuba, Japan. The hiPSCs were maintained on mouse embryonic fibroblasts (MEF) in StemFit Basic 03 media (Ajinomoto Co, Japan) with 5 ng/ml fibroblast growth factor-2 (FGF2) (Katayama Chemical Industries, Japan). After 3–4 passages on MEF, hiPSCs were transferred to feeder-free conditions in dishes coated with Matrigel matrix (Corning, NY) with StemFit Basic 03 media and 5 ng/ml FGF2. The human pancreatic cancer cell line BxPC3 cells (ATCC, VA) were cultured in RPMI-1640 (Wako, Japan) containing 10% fetal bovine serum (FBS) (Thermo Fisher Scientific, MA). When the cells reached 70–80% confluence, the media were changed to RPMI-1640 containing 5% knockout serum replacement (Thermo Fisher Scientific, MA). After 48 h, the conditioned media was collected and centrifuged at 1000×*g* for 10 min, and the supernatants were then filtered through 0.45 μm filters (Sartorius, Göttingen, Germany). For the conversion, the hiPSCs were passaged to new Matrigel coated dishes with StemFit Basic 03 media and, the media were changed after 24 h to StemFit Basic 03 with 50% of CM and 10 μM AZD6244 (Selleck, Japan), 1 μM CHIR99021, 1000 U/mL leukemia inhibitory factor (Merck Millipore, MA) and 5 ng/ml FGF2. Half of the media was changed daily and when cells reached 70–80% confluence, they were passaged to new dishes. The conversion process was continued up to one month. The hiPSCs in day 0 and after four weeks of conversion were cultured on gelatin coated dishes in DMEM media (Wako, Japan) with 15% FBS. The primary cultures of cells from tumors were prepared as same as described previously (Hassan et al. 2022). After digestion, 3 ml of StemFit Basic 03 medium with 10% FBS was added to stop digestion. The cell suspension was then centrifuged at 4000×*g* for 5 min, the supernatant was discarded, cell pellets were resuspended in 2 ml of StemFit Basic 03 medium, and cells were then seeded into Matrigel coated dishes at a density of 1 × 10^6^ cells/dish in StemFit Basic 03 medium. Blood samples were taken after obtained informed consent.

### Sphere formation assay

To evaluate the spheroid forming ability of cells, 1000 cells/well were seeded into 24-well ultra-low attachment plates (Sumitomo Bakelite, Japan) with StemFit Basic 03 medium. The plates were incubated at 37 °C for 10 days and then spheroids were photographed using an IX81 inverted microscope (Olympus, Japan).

### Flow cytometry

One million cells were washed with cold PBS and incubated for 20 min on ice with a blocking buffer consisting of 0.2% BSA (Miltenyi Biotec, Germany) in PBS. Then, cells were incubated with primary antibodies; anti-CD44 monoclonal antibody (IM7) (Thermo Fisher Scientific, MA), anti-CD24 monoclonal antibody (SN3) (Thermo Fisher Scientific, MA), anti- EpCAM monoclonal antibody (E8Q1Z) (Cell Signaling Technology, MA), and anti-CD133 monoclonal antibody (A8N6N) (Cell Signaling Technology, MA). After incubation, cells were washed with PBS and incubated with secondary antibodies; goat anti-rabbit IgG (H+L) Alexa Fluor® 647 (Thermo Fisher Scientific, MA), goat anti-rat (IgG) PE (Abcam, Cambridge, United Kingdom) and goat anti-mouse IgG (H+L) Alexa Fluor® 488 (Thermo Fisher Scientific, MA) on ice for 20 min. Then, cells were washed and run on Accuri C6 Plus flow cytometer (BD Biosciences, NJ). The obtained data was analyzed with Flowjo software (FlowJo, LLC, ORE).

### Animal experiments

The NOD-SCID mice were purchased from Charles River, Japan and randomly divided into groups of three mice in each. The hiPSCs, hiBxcm1 and hiBxcm2 cells, were passaged, counted and 2 × 10^6^ cells were prepared and injected into pancreases of mice. Mice were sacrificed after 5 weeks of injections and the tumors were excised. Details for animal experiments were in our previous report (Hassan et al. 2022). For serial transplantation of tumor tissues, the 4-week-old Balb/c nu/nu female mice (Charles River, Japan) were anesthetized for surgery. Then, a small cut in the skin was made, 3–4 mm tumor tissue pieces were prepared from tumors developed in pancreas of NOD-SCID mice, washed with PBS, transplanted into the cut area and skin was closed with sutures. Two months after transplantation, mice were sacrificed, and the tumors were excised. The animal experiments were conducted according to Okayama University guidelines under the approval No. 2020382 and 2020631 from the ethics committee for animal experiments of Okayama University.

### Histopathology

Tumor tissues fixed in formalin and embedded in paraffin were sectioned by a microtome RM2255 (Leica, Germany). For the nuclear/cytoplasm staining, the sections were stained with hematoxylin and eosin Y (Sigma-Aldrich, MO).

### Tube-formation assay

Cells were suspended in 500 μl EBM2 media supplemented with human epidermal growth factor (EGF; 5 ng/mL), vascular endothelial growth factor (VEGF; 0.5 ng/mL), R3-insulin like growth factor-1 (20 ng/mL), ascorbic acid (1 μg/mL), hydrocortisone (0.2 μg/mL), human basic fibroblast growth factor (bFGF; 10 ng/mL), heparin (22.5 μg/mL) (Lonza, Switzerland) and 2% FBS, and were seeded at a density of 7.5 × 10^4^ cells/well in 12-well plates coated with Matrigel. Twenty-four hours after seeding, tube structures formed by the cells were photographed with an IX81 inverted microscope.

### RNA-seq

Total RNA was extracted with TRIzol RNA isolation reagents (Life Technologies, CA, USA) and treated with DNase I (Promega, WI). Samples were subjected to sequencing with Novaseq6000 (Illumina, CA) and the obtained data were analyzed with Galaxy <usegalaxy.org> as descripted previously (Hassan et al. 2022). Briefly, differential expressed genes were identified with Cuffdiff tool and used as inputs to detect enriched pathways from Kyoto Encyclopedia of Genes and Genomes (KEGG) database using DAVID Bioinformatics Resources (Huang et al. 2009a, b). The integrated Differential Expression and Pathway analysis (iDEP) < http://bioinformatics.sdstate.edu/idep93/ > was used to generate the heat map and volcano plots (Ge et al. 2018).

### Immunofluorescence

Round coverslips (18 mm, Matsunami, Japan) were inserted into wells of 24-well plates, coated with Matrigel, and the cells were seeded at a density of 4 × 10^4^ cells/well. After 24 h, cells were fixed, blocked, permeabilized and incubated with anti-CD44 monoclonal antibody, anti-CD24 monoclonal antibody (Thermo Fisher Scientific, MA), anti-CD133 monoclonal antibody, anti-NANOG monoclonal antibody, anti-ALDH1A1 monoclonal antibody (Cell Signaling Technology, MA) or anti-OCT3/4 monoclonal antibody (Santa Cruz Biotechnology, TX). Cells were then incubated with PE labeled anti-rat goat IgG, Alexa Fluor 488 labeled anti-mouse goat IgG, or Alexa Fluor 555 labeled anti-rabbit goat IgG (Thermo Fisher Scientific, MA). The VECTASHIELD Mounting Medium (Vector Labs, CA) was used to mount coverslips and slides were photographed using an FSX100 fluorescent microscope (Olympus, Japan).

### Western blotting

Total protein was separated by sodium dodecyl sulfate polyacrylamide gel electrophoresis and then transferred to polyvinylidene fluoride membrane (Millipore Sigma, MA). After blocking with 5% skim milk, membrane was incubated overnight at 4 °C with primary antibodies against Erk1/2, phospho-Erk1/2 (Thr202/Tyr204), Akt, phospho-Akt (Ser473) (Cell Signaling Technology, MA) and β-actin (Wako, Japan). Then, membrane was incubated with either anti-mouse or anti-rabbit HRP–conjugated secondary antibodies, washed and visualized with the Western Lightning Plus ECL Substrate (Perkin Elmer, MA).

### Statistical analysis

The Graphpad prism 9 software (GraphPad Software, SD) was used to analyze data. Each data was presented as mean ± standard deviation and two-tailed t-test was used to assess the significance. The *p *value ≤ 0.05 was considered significant.

## Results

### BxPC3 cell’s CM with MEK and GSK-3β inhibitors converted hiPSCs into a new cell phenotype

**Fig. 1:**
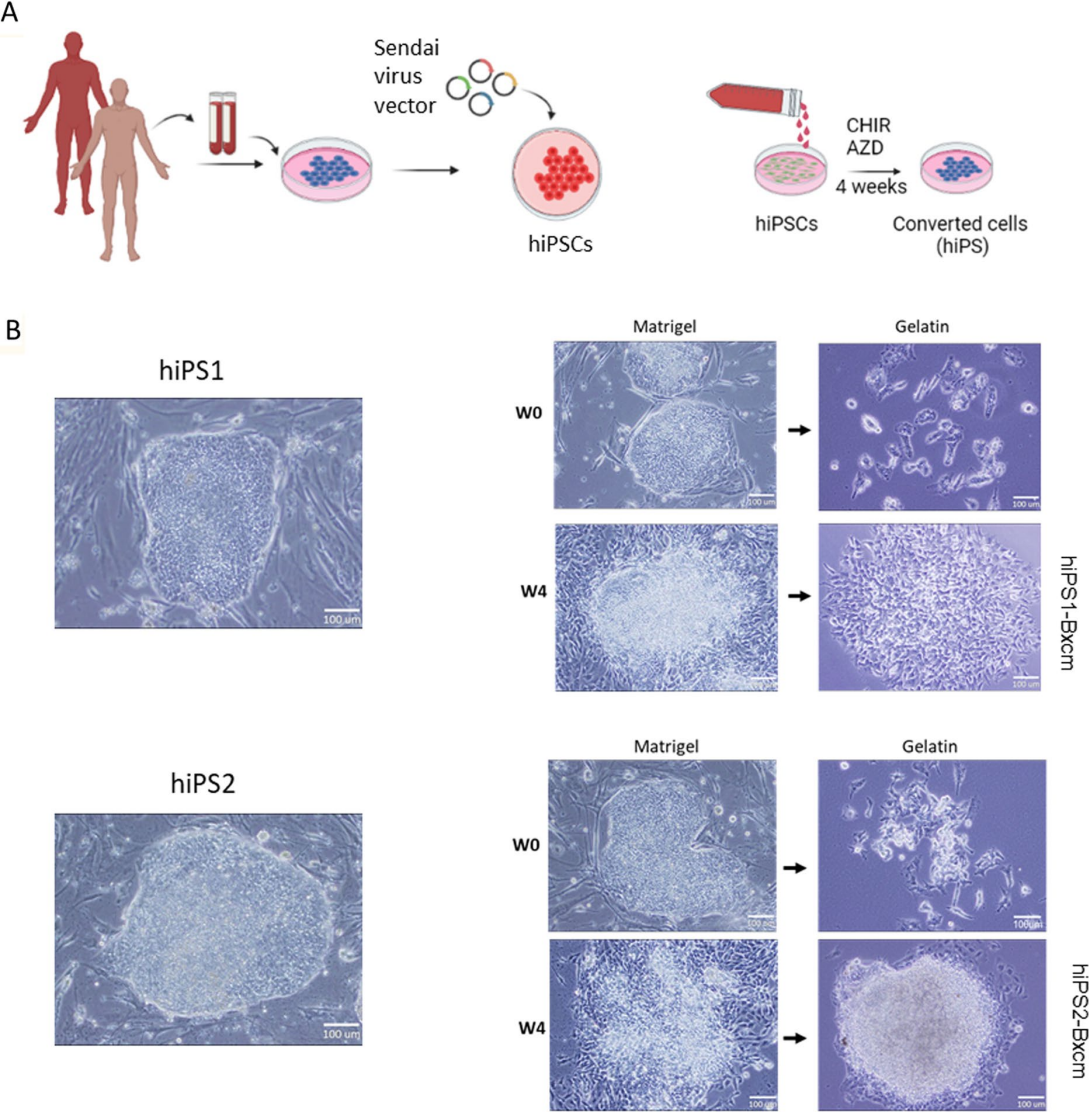
Conversion of hiPSCs with CM, AZD6244 and CHIR99021. **A** A schematic of the study. The hiPSCs were reprogrammed from monocytes and were cultured in media containing CM, CHIR99021 and AZD6244 for 4 weeks. **B** Representative images of established hiPSCs showing typical hiPSC colonies and images of cells cultured on either Matrigel, or gelation-coated dished at the beginning of conversion (W0) and after 4 weeks of conversion (W4) for both hiPS1 and hiPS2 cells

The iPSC technology has an advantage of providing pluripotent stem cells for adult individual. We firstly prepared two different hiPSCs from monocytes of two healthy volunteers. The colonies of hiPSC were cultured on the feeder layer and showed a typical morphology with compact cells (Fig. 1A, B). The cell lines were designated as hiPS1 and hiPS2. The hiPSCs are typically being cultured in culture vessels coated with laminin or Matrigel matrix with serum-free media to maintain stemness. The maintenance of stemness and survival of hiPSCs were not accomplished on uncoated or gelatin-coated culture vessels in the media supplemented with FBS because these conditions promote cell differentiation. When hiPS1 and hiPS2 cells were cultured on gelatin-coated dishes in DMEM media with 15% FBS, cells began to differentiate, stopped proliferation, and did not survive beyond one week (Fig. 1B). We demonstrated that CM from different cells of human cancer cell lines was rich in growth factors and cytokines that could convert mouse iPSCs into CSCs (Afify et al. 2020; Calle et al. 2016; Chen et al. 2012; Hassan et al. 2022). Further, we showed that microenvironment of pancreatic cancer cell line BxPC3 cells converted mouse iPSCs into aggressive CSCs (Hassan et al. 2022). In this context, microenvironments of different pancreatic cancer cell line cells could convert mouse iPSCs into different CSCs with different plasticity. Therefore, we employed the CM from BxPC3 cells in this study to mimic the microenvironment of tumor initiation. The cells cultured in 50% CM of BxPC3 cells with CHIR99021 and AZD6244 maintained cell proliferation up to one month. These cells changed their survival character and could sustain proliferation and survival when cultured in gelatin-coated dishes in FBS supplemented media (Fig. 1B). These results indicate that cell phenotypes were changed when cultured in CM with two inhibitors. Cells converted from hiPS1 and hiPS2 cells were designated as hiPS1-Bxcm and hiPS2-Bxcm cells, respectively.

### Converted cells from hiPSCs demonstrated CSC characteristics

**Fig. 2:**
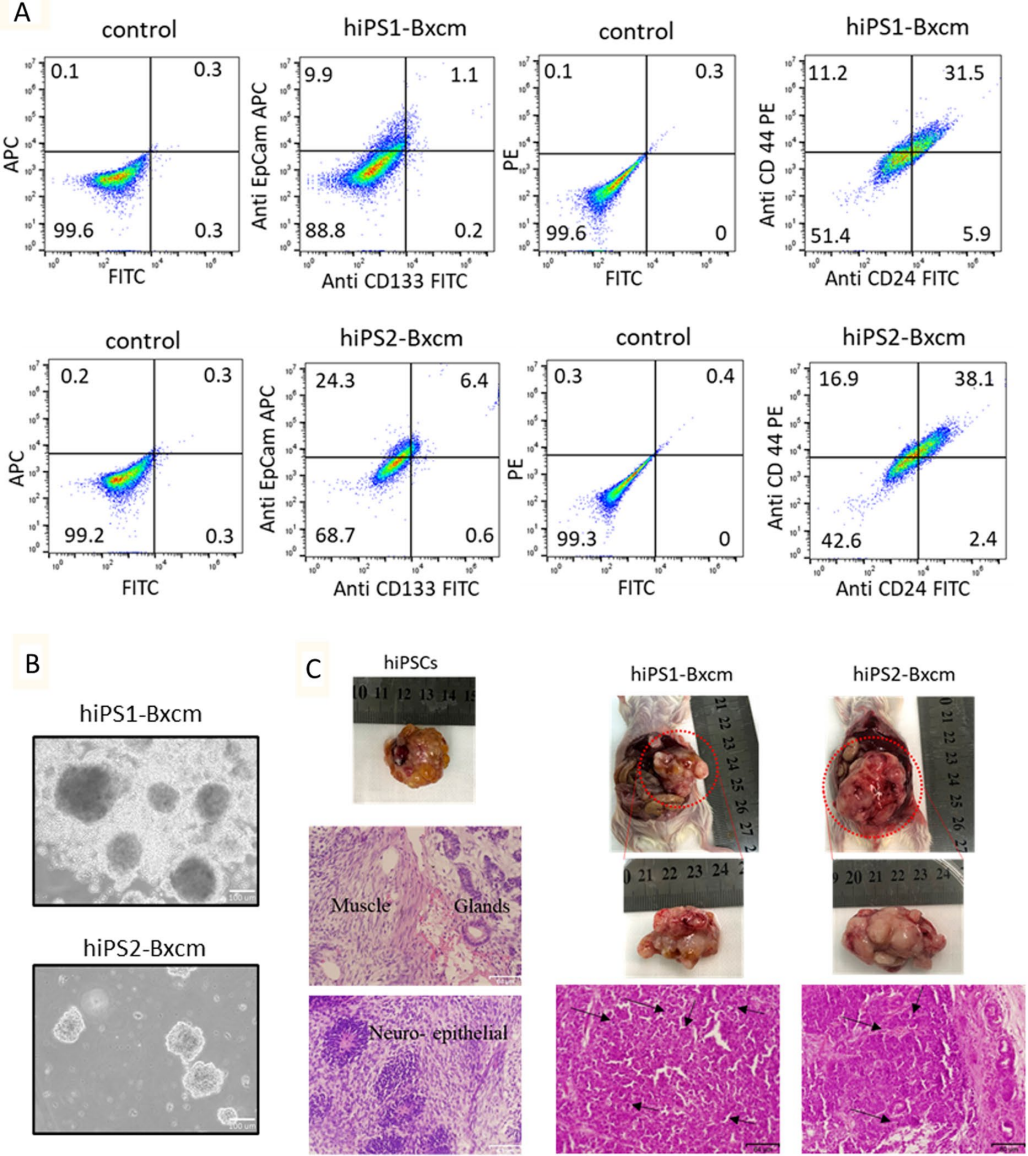
Self-renewal ability, expression of CSC markers and tumorgenicity of converted cells. **A** Representative flow cytometry plots of hiPS1-Bxcm cells (first row) and hiPS2-Bxcm cells (second row) assessed by anti-EpCAM Ab, Anti-CD133 Ab, anti-CD24 Ab, and anti-CD44 Ab. **B** Representative images of spheroids formed by hiPS1-Bxcm and hiPS2-Bxcm cells in low attachment culture conditions. **C** Representative images of mice and tumors developed by the injection of the hiPS1-Bxcm and hiPS2-Bxcm cells and tumor sections stained with hematoxylin and eosin. The hiPSC derived tumors showed teratoma phenotype with glands, neuroepithelial and muscle tissues. The hiPS1-Bxcm and hiPS2-Bxcm cell tumors exhibited malignant phenotypes with no teratoma. Arrows are cells with mitotic figures and nuclear atypia. Scale bars = 64 μm

The flow cytometric analysis showed that converted cells had subpopulations expressing CSC markers, CD133, EpCAM, CD24 and CD44. The percentages of cells positive for EpCAM were 11% and 30.7% while those for CD133 were 1.3% and 7% in hiPS1-Bxcm and hiPS2-Bxcm, respectively. The CD44 positive cells were 43.7% and 55% while CD24 positive cells were 37.4% and 40.5% in hiPS1-Bxcm and hiPS2-Bxcm, respectively (Fig. 2A). CSCs are known for their ability to form spheroids in low attachment culture conditions. The hiPSCs cultured in CM and two inhibitors maintained this ability and formed spheroids when cultured in low attachment conditions indicating the maintenance of self-renewal ability (Fig. 2B).

The ability of hiPS1-Bxcm and hiPS2-Bxcm cells to form malignant tumors was assessed by the injection of cells into the pancreases of NOD-SCID mice. The injected mice developed tumors in the pancreas and the histological analysis of the tumor sections showed malignant phenotypes. Sections had high nucleus to cytoplasm ratio, cells with severe nuclear atypia and mitotic figures while no benign teratoma phenotype was found in these sections (Fig. 2C). The tumors derived from injection of hiPSCs on the other hand showed teratoma phenotype as benign tumors. Sections of the tumors of hiPSCs had tissues deriving from three germ layers. The mesoderm-derived muscle tissues, ectoderm-derived neuroepithelial tissues, and endoderm-derived glands were detected in hiPSC derived tumors sections (Fig. 2C).

### Converted cells kept their tumorigenicity during serial transplantation and tumor primary cells exhibited CSC phenotype

**Fig. 3:**
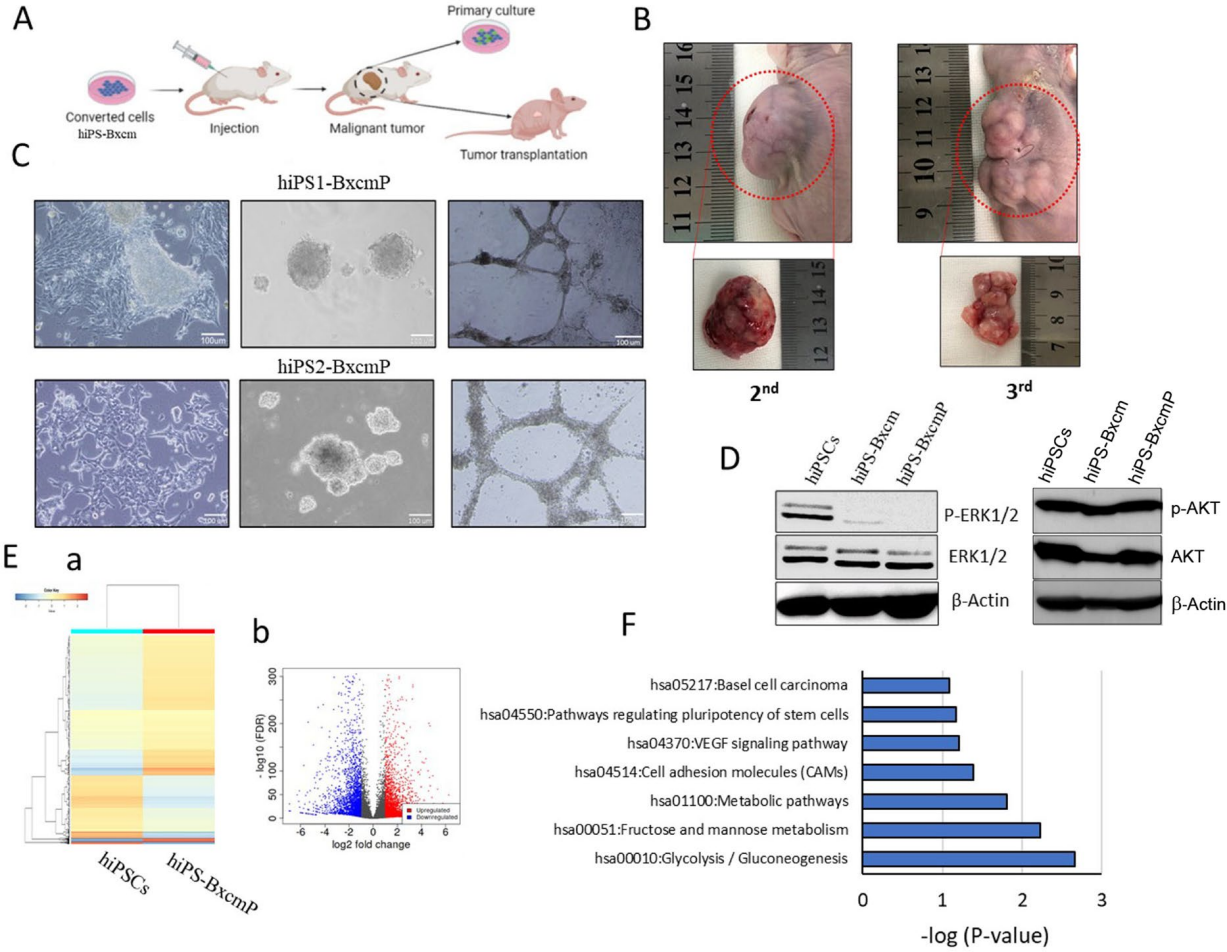
The tumorigenicity of serial transplantation of tumor tissues and top enriched pathways of CSCs converted from hiPSCs. **A** A schematic of preparation of primary cultures and tumor transplantation. **B** Representative images of mice and secondary (2nd) and tertiary (3rd) tumors formed by transplantation of tumor tissues of hiPS1-Bxcm tumors. **C** Representative images of cells isolated from primary culture of hiPS1-Bxcm tumors (hiPS1-BxcmP cells) and from hiPS2-Bxcm tumors (hiPS2-BxcmP cells), spheroid formed by hiPS1-BxcmP and hiPS2-BxcmP cells in low attachment culture conditions and tube-like structures formed by hiPS1-BxcmP and hiPS2-BxcmP cells. Scale bars = 100 μm. **D** Western blots of hiPSCs, induced CSCs (hiPS-Bxcm) and primary cells isolated from tumors (hiPS-BxcmP). **E** Transcriptome analysis of hiPS-BxcmP cells compared with hiPSCs. (*a*) A heat map of gene expression patterns of cells, (*b*) a volcano plot showing up and downregulated genes. **F** a group of top enriched pathways for upregulated genes in hiPS-BxcmP cells compared with hiPSCs

To investigate whether the tumor derived from hiPSCs treated with the CM of BxPC3 cells could maintain their tumorigenicity through serial transplantation or not, tumor tissues developed from converted cells were transplanted subcutaneously into nude mice (Fig. 3A). Tumor tissues grew and mice developed secondary and tertiary tumors indicating the long-term maintenance of tumorigenicity (Fig. 3B). The cells isolated from hiPS1-Bxcm tumors (hiPS1-BxcmP) and hiPS2-Bxcm tumors (hiPS2-BxcmP) formed spheroids when cultured in low attachment conditions indicating their self-renewal ability. Both types of cells also exhibited the ability to form tube-like structures when cultured on Matrigel with an endothelial basal medium (Fig. 3C). The western blotting results showed that phosphorylation of ERK1/2 was diminished in induced CSCs and primary cells (hiPS-BxcmP) indicating inhibition of MAPK cascade, while the presence of phosphorylated AKT indicated the activated PI3K pathway in both converted cells and primary cells (Fig. 3D). Next, we compared the gene expression pattern of hiPS1-BxcmP cells with that of hiPSCs using RNA-seq. The data was plotted as a heat map (Fig. 3E(a)). The differentially expressed genes (DEGs) were presented as volcano plots showing that different genes are up- and down-regulated in hiPS-BxcmP cells (Fig. 3E(b)). More than 180 genes were found specifically expressed in hiPS-BxcmP cells but not in hiPSCs (Supplementary Table 1). The KEGG pathway enrichment analysis of these genes showed that enriched pathways were including signaling pathways regulating pluripotency of stem cells and pathways in cancer (Supplementary Table 2). The KEGG analysis of all upregulated genes in hiPS-BxcmP cells showed that enriched pathways were basically related to tumorigenesis, angiogenesis, cell mobility, stemness and cell metabolism (Fig. 3F) (Supplementary Table 3).

**Fig. 4:**
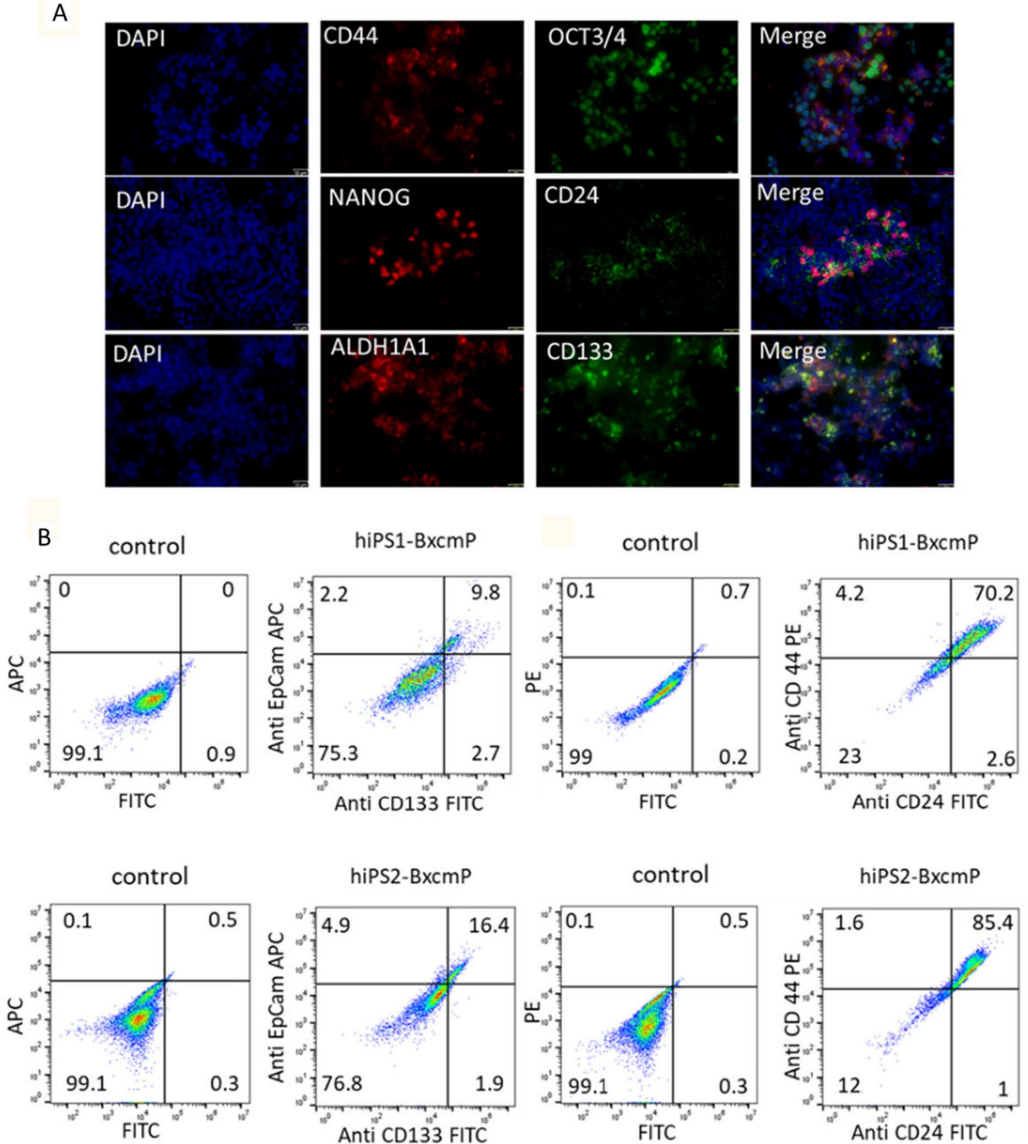
The expression of CSC markers in primary cells isolated from hiPS1-Bxcm and hiPS2-Bxcm tumors. **A** Representative images of immunofluorescence staining of hiPS1-BxcmP cells stained with CSC markers, CD133, CD24 and ALDH1A1, and stemness markers, OCT3/4 and NANOG. Scale bars = 32 μm. **B** flow cytometry plots of hiPS1-BxcmP cells (first row) and hiPS2-BxcmP cells (second row) assessed by anti-EpCAM Ab, Anti-CD133 Ab, anti-CD24 Ab, and anti-CD44 Ab

The immunofluorescence staining showed that primary cells were positive for stemness markers NANOG and OCT3/4 and CSC markers CD44 and ALDH1A1 (Fig. 4A). The flow cytometric analysis also showed that the percentages of cells double-positive for both CD133 and EpCAM were 9.8% and 16.4% in hiPS1-BxcmP and hiPS2-BxcmP cells, respectively, while the percentages of cells double-positive for both CD24 and CD44 were 70.2% and 85.4% in hiPS1-BxcmP and hiPS2-BxcmP cells, respectively (Fig. 4B).

Collectively, these data show that converted cells and that isolated from their tumors exhibited CSC characteristics including self-renewal, angiogenesis, and expression of CSC markers suggesting that hiPSCs were converted into CSCs when cultured in media with CM, AZD6244, and CHIR9902.

## Discussion

The conversion of iPSCs into CSCs will provide unique models for cancer research. Although the conversions of mouse iPSCs into CSCs are usually successful in our lab, those of human iPSCs have been less successful. The majority of the iPSC derived CSC models established with CM was from mouse cells (Afify et al. 2020; Calle et al. 2016; Chen et al. 2012; Du et al. 2020; Hassan et al. 2022; Hassan and Seno 2020). Here, the CM and two signaling inhibitors, CHIR99021 and AZD6244, were assessed to convert hiPSCs into CSCs. Cells treated with this combination changed their phenotype where they acquired CSC characteristics. The CM mimics the tumor initiation microenvironment and thought to trigger the conversion of iPSCs into CSCs. The blockade of both GSK3β and MEK seem to be critical to maintain stemness of converted cells. The PI3K/AKT and MEK/ERK pathways regulate cell stemness. The role of crosstalk between MEK/ERK, PI3K/AKT and WNT pathways in CSCs is not clear yet and the results from this study could give some insight into the conversion of normal human stem cells into CSCs under pancreatic microenvironment (Sim et al. 2017). Here, two hiPSC cell lines, reprogrammed from two different healthy volunteers, were converted into CSCs. The resultant cells from conversion and those isolated from tumors exhibited different expression levels of CSC markers. The percentage of cells positive for both EpCAM and CD133 or positive for both CD44 and CD24 were different between hiPS1-Bxcm and hiPS2-Bxcm cells. The primary cells isolated from tumors, hiPS1-BxcmP and hiPS2-BxcmP cells, also had different percentages of cells positive for previous markers. Even with the same conversion conditions, the resultant cells were different between two cell lines. These differences could be due to the differences in epigenetic and genetic profiles of obtained cells. Patients respond to cancer treatments differently depending on their genetic and epigenetic profiles. The data here could explain these differences according to susceptibility of cells to be converted into CSCs and suggest that MEK/ERK pathway inhibition along with activation of PI3K/ AKT pathway represented by phosphorylation of AKT may play roles in the conversion process of stem cells to CSCs. Moreover, the CSC stemness could be maintained through MEK/ERK and WNT pathway crosstalk. The simultaneous inhibition of GSK-3β and MEK/ERK coupled with CM stimulation could convert hiPSCs into CSCs indicating the dual inhibition might help maintain cell stemness by activating Akt during the conversion process. Liao et al*.* also reported that immortalized human mammary epithelial cells could be converted into cancer cells with stemness characteristics by MEK and GSK3 inhibitors (Liao et al. 2018). The inhibitors were suggested to co-stiumale cancer cells to generate CSCs. Our data consist with this report that MEK and GSK3 inhibitors could maintain stemness. However, we used iPSCs without genetic modification while Liao et al*.* used immortalized human mammary cells. Moreover, here we used the AZD6244 as MEK1 inhibitor which we previously reported that it was more effective in the conversion of miPSCs into CSCs than MEK1/2 inhibtors (Hassan et al. 2021).

The advantage of CSC models derived from iPSCs presented here will be the availability in studying the tumorigenesis process without genetic manipulation which allow creating different plastic CSCs. Further research is required to explore the exact mechanism and factors involved in the conversion process and the specific conditions required to convert hiPSCs into CSCs that mimic CSCs derived from patients with tissue-specificity. Therefore, these data could be further investigated in the future to understand CSC induction and the mechanisms of maintenance to selectively prevent their occurrence and to develop more efficient treatments.

## Supplementary Information

Below is the link to the electronic supplementary material.

Supplementary file1 (XLSX 2755 kb)

Supplementary file2 (XLSX 10 kb)

Supplementary file3 (XLSX 13 kb)

## Data Availability

All dataset used in this study are included in the paper and its supplementary files.
